# Gene-Transformation-Induced Changes in Chemical Functional Group Features and Molecular Structure Conformation in *Alfalfa* Plants Co-Expressing *Lc*-*bHLH* and *C1-MYB* Transcriptive Flavanoid Regulatory Genes: Effects of Single-Gene and Two-Gene Insertion

**DOI:** 10.3390/ijms18030664

**Published:** 2017-03-20

**Authors:** Ravindra G. Heendeniya, Peiqiang Yu

**Affiliations:** Department of Animal and Poultry Science, College of Agriculture and Bioresources, University of Saskatchewan, Saskatoon, SK S7N5A8, Canada; rgv863@mail.usask.ca

**Keywords:** molecular structure, function groups, gene transformation, nutrition and structure interaction, *alfalfa* plant, molecular spectroscopy

## Abstract

*Alfalfa* (*Medicago sativa* L.) genotypes transformed with *Lc-bHLH* and *Lc* transcription genes were developed with the intention of stimulating proanthocyanidin synthesis in the aerial parts of the plant. To our knowledge, there are no studies on the effect of single-gene and two-gene transformation on chemical functional groups and molecular structure changes in these plants. The objective of this study was to use advanced molecular spectroscopy with multivariate chemometrics to determine chemical functional group intensity and molecular structure changes in *alfalfa* plants when co-expressing *Lc-bHLH* and *C1-MYB* transcriptive flavanoid regulatory genes in comparison with non-transgenic (NT) and AC Grazeland (ACGL) genotypes. The results showed that compared to NT genotype, the presence of double genes (*Lc* and *C1*) increased ratios of both the area and peak height of protein structural Amide I/II and the height ratio of α-helix to β-sheet. In carbohydrate-related spectral analysis, the double gene-transformed *alfalfa* genotypes exhibited lower peak heights at 1370, 1240, 1153, and 1020 cm^−1^ compared to the NT genotype. Furthermore, the effect of double gene transformation on carbohydrate molecular structure was clearly revealed in the principal component analysis of the spectra. In conclusion, single or double transformation of *Lc* and *C1* genes resulted in changing functional groups and molecular structure related to proteins and carbohydrates compared to the NT *alfalfa* genotype. The current study provided molecular structural information on the transgenic *alfalfa* plants and provided an insight into the impact of transgenes on protein and carbohydrate properties and their molecular structure’s changes.

## 1. Introduction

The *alfalfa* plant is the “queen” of foraging due to its high nutritive value, particularly its high level of protein. However, extremely high soluble protein content in *alfalfa* plants causes major issues when ruminant livestock graze on *alfalfa* pasture, which include bloating causing animal death, N-to-energy unsynchronization, and nutrients being under-utilized [1,2]. How can we solve these issues? It has been found that the soluble protein in *alfalfa* plants can bind with proanthocyanidin (PA) to form a complex. This complex can prevent protein from being degraded in the rumen but shifts protein from the rumen to small intestine. In this way, the three major issues can be solved. But how can we make *alfalfa* plants produce PA?

Recent attempts to engineer proanthocyanidin (PA) synthesis in *alfalfa* by transformation of transcription factors such as *Lc-bHLH* [3], LAP1-MYB, PAP1-MYB [4] and more recently TaMYB14 [5] have had varying degrees of success in improving *alfalfa* protein quality. However, the effects of these single-gene and double-gene insertion/transformation on alfalfa’s molecular structure feature changes and chemical functional groups that related to protein utilization and digestion have not been studied.

Vibrational molecular spectroscopy-Fourier transform infrared spectroscopy (VMS-Ft/IR) has been used in the past as a non-destructive analytical technique to study molecular structures associated with nutritive value of plant material [6,7,8,9,10].

Recently, the *alfalfa* genotype with both *Lc-bHLH* and *C1-MYB* co-expressed plants became available. To our knowledge, there are no studies on the effects of single-gene and two-gene transformation on chemical functional groups and molecular structure changes in these transgenic plants. The objective of this study was to determine chemical functional group intensity and molecular structure changes in *alfalfa* forage plants that co-express *Lc-bHLH* and *C1-MYB* transcriptive flavanoid regulatory genes.

## 2. Results and Discussion

### 2.1. Effect of Single-Gene and Double-Gene Insertion/Transformation on Changes Chemical Functional Groups and Molecular Structure Related to Protein Properties

In univariate analyses, there were no significant differences (*p* > 0.05) in overall comparison of Amide-II and β-sheet spectral heights among different *alfalfa* genotypes (Table 1). The *Lc1* genotype has exhibited the lowest parameters associated with both primary and secondary protein molecular structures i.e., Amide-I height (0.024) and area (1.261), Amide-II area (1.729), total Amide area (2.990), α-helix height (0.023) and α-helix/β-sheet ratio (1.109). The comparison with parent non-transgenic (NT) *alfalfa* shows that both the intensity (spectral height) and extent (band area) of Amide-II are lower in transformed *alfalfa*, so it has a significantly higher Amide-I/II ratio. There was also a significantly higher Amide-I/II ratio in double gene *alfalfa* compared to NT *alfalfa*. As stated before, Amide-I and Amide-II vibrations are governed by stretching of C=O and in-plane bending of N–H, respectively [11]. The Amide-II region is also associated with protein conformation [12]. Taken together, these results indicate that gene transformation has changed the intrinsic structural make-up of proteins and protein conformity. However, since α-helix and β-sheet spectral data (heights and ratios) that represent protein secondary structures have not shown significant differences between NT and transformed *alfalfa*, gene transformation may not significantly influence the protein degradation. Similar results have been reported by Yu et al. [12], in their protein molecular study on *Lc-*transformed *alfalfa* using synchrotron radiation VMS-Ft/IR.

**Figure 1:**
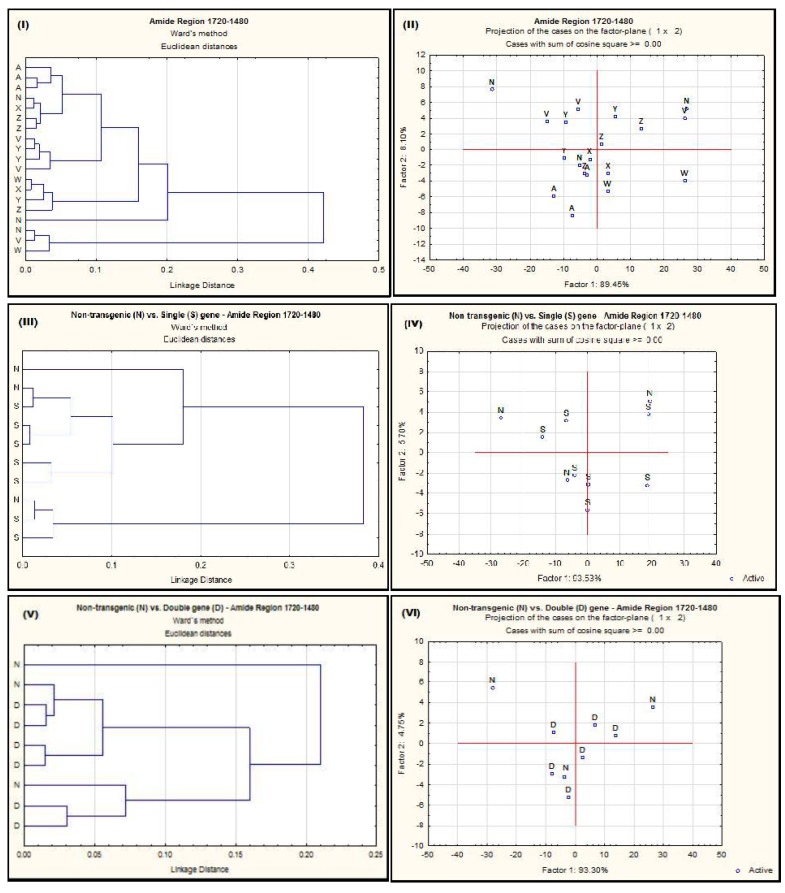
Cluster (**I**,**III**,**V**) and principal component (**II**,**IV**,**VI**) analyses of spectrum detected with VMS-Ft/IR in the amide region obtained from different *alfalfa* populations. A = AC-Grazeland; N = Non-transformed parent plant; V = Single transformed *C1*; W = Single transformed *Lc1*; X = Single transformed *Lc3*; Y = Double transformed *Lc1xC1*; Z = Double transformed *Lc3xC1*; S = single-gene transformed; D = double gene transformed.

In multivariate analysis, Agglomerative hierarchical cluster analysis (AHCA) and Principal Component Analysis (PCA) methods were used to discriminate the IR spectrum in the Amide region 1720–1480 cm^−1^. In this region the ACGL genotype clearly separated from other genotypes in AHCA (Figure 1I). The Amide region could not be separated for NT vs. single-gene or NT vs. double gene-transformed *alfalfa* populations by either AHCA or PCA (Figure 1III–VI). As mentioned before, there are no studies on the effect of single-gene and two-gene insertion on the molecular structure changes in these trans-plants (*C1*, *Lc1*, *Lc3*, *Lc1C1* and *Lc3C1*). No comparison or discussion with published results could be conducted in this section.

### 2.2. Effect of Single-Gene and Double-Gene Insertion/Transformation on Changes in Chemical Functional Groups and Molecular Structure with Regard to Carbohydrate Properties

The spectral features of structural carbohydrate (StCHO), cellulosic compounds and total carbohydrates (TCHO) are shown in Table 2. In the overall comparison of StCHO, there was no significant difference among the different *alfalfa* genotypes in relation to peak 3 intensities. However, intensities of peak 1 and 2 as well as total StCHO areas tended to be different (*p* < 0.10) among genotypes. The contrast analysis has shown that peak 1 intensity in ACGL was significantly lower (*p* < 0.05) than both single and double gene-transformed *alfalfa* genotypes. The peak 2 intensities of all the transgenic *alfalfa* were significantly lower (*p* < 0.05) than that of NT *alfalfa*. Probably due to lower absorption heights at peaks 1 and 2, the total structural carbohydrate areas in transformed *alfalfa* were lower than NT *alfalfa*. There was no significant difference (*p* > 0.05) in spectral data of cellulosic compounds or total area of the TCHO region among *alfalfa* populations. However, significantly higher intensities of peak 1 (*p* < 0.05) and peak 3 (*p* < 0.01) for the TCHO spectral region were observed in NT *alfalfa* compared to *Lc3C1* genotype. In the TCHO region, significantly, the highest genotype variations (*p* < 0.01) were observed under the peak 3 intensity and peak 3 area with the lowest peak 3 intensity and extent recorded with the ACGL genotype. The spectral data are influenced by both quantitative and qualitative features of a molecule. The peaks (1 and 3) at wave numbers ~1410 cm^−1^ and ~1240 cm^−1^ are closer to typical wave numbers of β-glucans (~1420 cm^−1^) and hemicellulose (1246 cm^−1^) respectively [13,14,15]. Since there was no significant difference in the contents of StCHO (represented by NDF and ADF), the differences observed in relation to peaks and total areas of StCHO spectral region among NT and transgenic *alfalfa* imply that gene transformation has an effect on the strength and polarity of vibrating bonds associated with StCHO. On the other hand, peak 3 at 1020 cm^−1^ of the TCHO region represents non-structural CHO such as starch [15]. The pattern of variation in peak 3 intensity and extent were similar to the starch content variations among the genotypes, indicating that the variations in TCHO peak 3 profiles in this experiment are influenced mainly by the respective starch contents.

**Figure 2:**
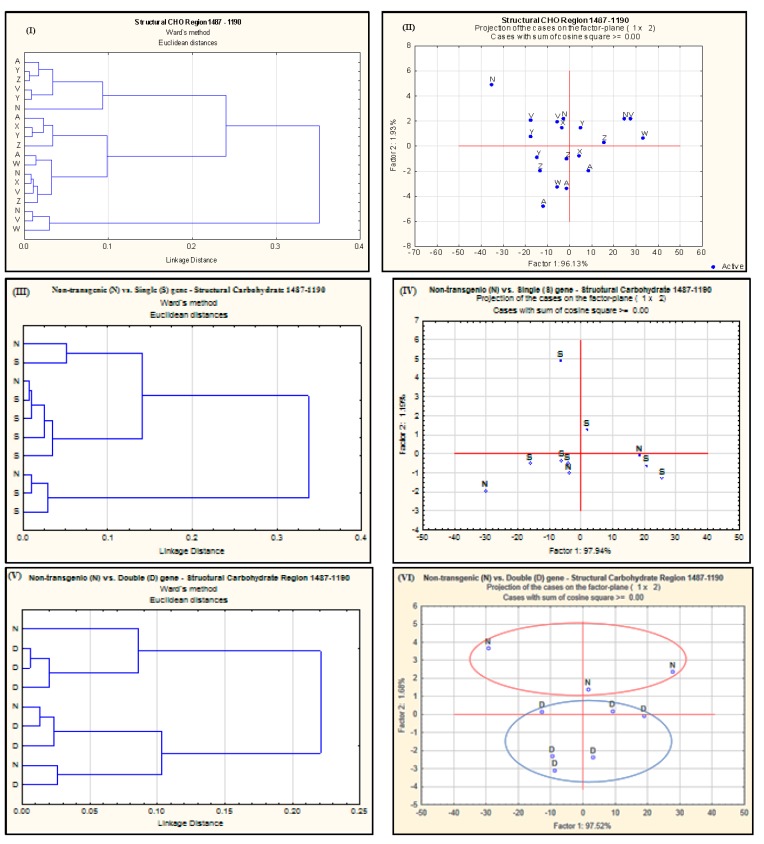
Cluster (**I**,**III**,**V**) and principal component (**II**,**IV**,**VI**) analyses of spectrum detected with VMS-Ft/IR in the structural carbohydrate region obtained from different *alfalfa* populations. A = AC-Grazeland; N = Non-transformed parent plant; V = Single transformed *C1*; W = Single transformed *Lc1*; X = Single transformed *Lc3*; Y = Double transformed *Lc1xC1*; Z = Double transformed *Lc3xC1*; S = single-gene transformed; D = double-gene transformed.

**Figure 3:**
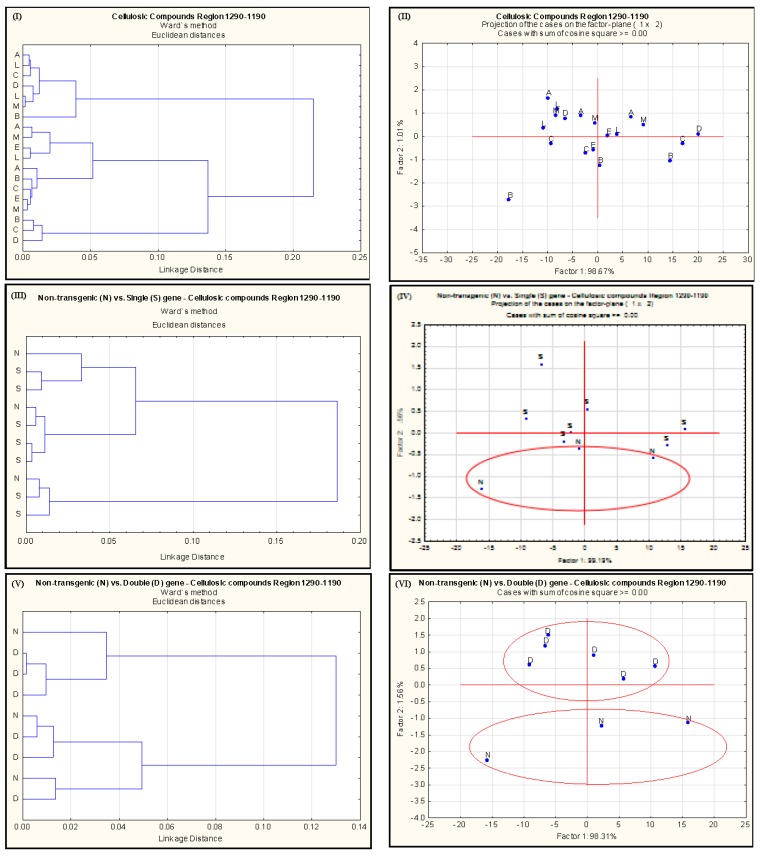
Cluster (**I**,**III**,**V**) and principal component (**II**,**IV**,**VI**) analyses of spectrum detected with VMS-Ft/IR in the cellulosic compound region obtained from different *alfalfa* populations. A = AC-Grazeland; B = Non-transgenic parent plant; C = Single transgenic *C1*; D = Single transgenic *Lc1*; E = Single transgenic *Lc3*; L = Double transgenic *Lc1xC1*; M = Double transgenic *Lc3xC1*; N = Non-transformed; S = single-gene transformed; D = double-gene transformed.

**Figure 4:**
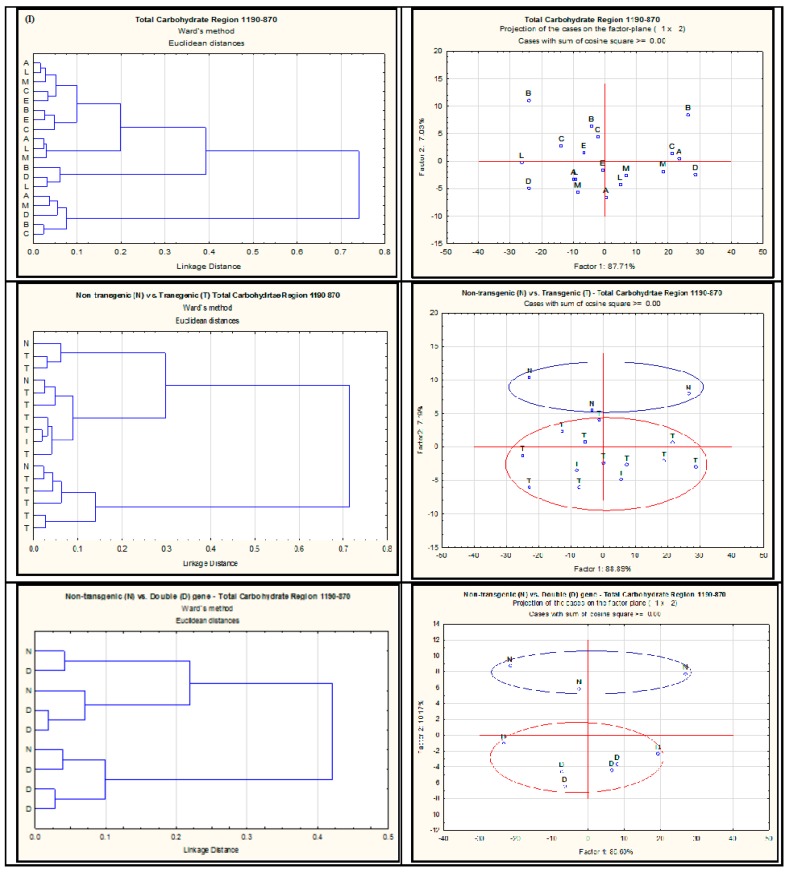
Cluster (**I**,**III**,**V**) and principal component (**II**,**IV**,**VI**) analyses of spectrum detected with VMS-Ft/IR in the total carbohydrate region obtained from different *alfalfa* populations. A = AC-Grazeland; B = Non-transgenic parent plant; C = Single transgenic *C1*; D = Single transgenic *Lc1*; E = Single transgenic *Lc3*; L = Double transgenic *Lc1xC1*; M = Double transgenic *Lc3xC1*. N = Non-transformed; S = single-gene transformed; D = double-gene transformed.

The multivariate analyses of the IR spectrum related to different carbohydrate regions are shown in Figure 2, Figure 3 and Figure 4. The cluster analysis has not revealed any discrimination of spectral data related to StCHO region (Figure 2I,III,V) of the IR spectrum among different *alfalfa* populations. In PCA however, there was a clear discrimination between NT and double gene-transformed *alfalfa* populations (Figure 2VI). This reflects the differences observed in the total areas of the StCHO region in these *alfalfa* populations (Table 2), probably as a result of differences at peak 2 (~1370 cm^−1^) and peak 3 (~1240 cm^−1^). The PCA of cellulosic region further reveals a clear discrimination of NT from both the single-gene and double-gene-transformed *alfalfa* populations (Figure 3IV,VI). Since there was no significant difference in NDF or ADF between NT and transgenic *alfalfa* (*p* > 0.05), the NT vs. double gene discrimination observed in PCA can be attributed to qualitative or “molecular–structural” differences in StCHO components. The NT *alfalfa* populations were also discriminated from all the transgenic *alfalfa* in the TCHO region (Figure 4III), particularly from double gene-transformed *alfalfa*. As stated before, the TCHO region mainly represents the non-structural carbohydrates, which include starch in the plant material. The discrimination of NT from transgenic *alfalfa* in PCA can be attributed to the differences in both content and molecular structure of starch.

Once again, there is no study on the effect of single-gene and two-gene insertion (*C1*, *Lc1*, *Lc3*, *Lc1C1* and *Lc3C1*) on the molecular structure changes in these trans-plants. Therefore no comparison and discussion could be made regarding published results.

## 3. Materials and Methods

### 3.1. Transgenic Alfalfa Plant Material

All the plant material was grown and maintained at the Saskatoon Research Center, Agriculture and Agri-Food Canada. The single-gene (*Lc1*, *Lc3* and *C1*) and double-gene (*Lc1C1* and *Lc3C1*) transformed *alfalfa* genotypes along with non-transformed parent genotype (NT) were grown from seeds initially in the greenhouse. The non-transgenic AC Grazeland (ACGL) plants for this experiment were propagated vegetatively from existing plant stock maintained at the greenhouse. The plants were tested for the presence of respective transgenes by PCR, repotted and transferred into a growth chamber where the light intensity, duration of light/dark and the temperature were maintained throughout the trial period at 550 µE·m^−2^·S^−1^, 16 h/8 h and 22 °C respectively. The *alfalfa* plants were harvested with shears 5 cm above soil level, when the plants reached late-bud stage [16]. The harvested material were immediately frozen at −20 °C and subsequently freeze-dried.

### 3.2. Molecular Spectroscopy

The molecular structures of samples were analyzed using a JASCO Fourier-transformed infrared vibration spectroscopy (VMS-Ft/IR; JASCO Corporation, Tokyo, Japan, model-4200) with a ceramic IR light source. The VMS-Ft/IR consist of a deuterated l-alanine doped triglycine sulfate detector armed with a MIRacle^TM^ attenuated total reflectance accessory module and equipped with a ZnSe crystal and pressure clamp (PIKE Technologies, Madison, WI, USA).

The transgenic and non-transgenic bio-forage samples were also tested using advanced synchrotron radiation-based Fourier transform IR microspectroscopy (SR-IMS) at the National Synchrotron Light Source in Brookhaven National Laboratory (NSLS-BNL, Upton, NY, USA) and Advanced Light Source in Berkeley National Laboratory (ALS-BNL, Berkeley, CA, USA) before using the VMS-Ft/IR approach.

### 3.3. Univariate Molecular Structure Spectral Processing and Analyses

JASCO spectra-manager II software was used to generate the spectra in the infrared range of 4000–800 cm^−1^ at a resolution of 4 cm^−1^. After noise elimination by JASCO spectra-manager™ II software (Tokyo, Japan), functional spectral bands were assigned for carbohydrates and proteins as per previous studies [12,13,14,17] and identified using software OMNIC version 8.2 (Thermo Fisher Scientific, Madison, WI, USA).

There are two primary frequency bands, i.e., structural Amide I (ca. 1700–1600 cm^−1^) and Amide II (1560–1500 cm^−1^) identifiable in the infrared spectrum resulting from vibration of C–O, C–N and N–H groups within the protein molecules. The Amide I band arising from stretching vibration of mainly the C–O group (80%) and to a lesser extent (20%) by C–N group shows a peak at ca. 1655 cm^−1^ while Amide II band shows a peak at ca. 1550 cm^−1^ due to bending vibration of N–H group (60%) and stretching vibration of C–N group (40%). The protein secondary structures α-helix and β-sheet peaks are located within the Amide I band and were identified using the second derivative function of OMNIC software [11].

The infrared spectrum of total carbohydrates (TCHO) that lies between wave numbers ca. 1180–900 cm^−1^, arises from the stretching vibration of C–O and C–C and the deformation of C–OH. The spectral band of structural carbohydrates (StCHO; cellulose, hemicellulose and β-glucans) lies typically between ca. 1485–1188 cm^−1^ with peaks at ca. 1410, 1370 and 1240 cm^−1^ [18].

### 3.4. Multivariate Molecular Spectral Analysis on Intrinsic Structure Changes by Gene Inserting

Two different multivariate methods were employed in the current study to perform multivariate spectral analysis using Statistical 8.0 (StatSoft Inc., Tulsa, OK, USA). Agglomerative hierarchical cluster analysis (CLA), which uses Ward’s Algorithm method without parameterization for clusting, presents results as dendrograms [19,20,21]. Principal Component Analysis (PCA), which is the other multivariate analysis method, transforms all interrelated variances into new uncorrelated variances called principles components (PCs) [19,20,21]. The result of PCA is presented as a scatter plot using two main PCs, which took more than 95% of variance, in form of PC1 vs. PC2.

### 3.5. Statistical Analyses

The spectral intensity data were analyzed by PROC MIXED of SAS (2003) 9.3 version according to statistical model:

$$Y_{ij}=\;\mu \;+\;P_{i}+{\;\varepsilon }_{ij}$$

where, *Y_ij_* is the dependent variable, μ is the overall mean, *P_i_* is the fixed effect of *alfalfa* population (*i* = 7; NT, ACGL, *C1*, *Lc1*, *Lc3*, *Lc1C1*, *Lc3C1*) and ε*_ij_* is the residual error.

The Tukey’s test was used for multiple population comparisons with letter groupings obtained using SAS pdmix800 macro [22]. The contrasts between means of different combinations of *alfalfa* populations were conducted using a statement from SAS. For all statistical analyses, significance was declared at *p* < 0.05 and tendency was declared at 0.05 < *p* < 0.10.

## 4. Conclusions

The results in this study showed that the transformation of *Lc* and *C1* genes have caused changes in the inherent molecular structures and chemical functional group intensity of both protein and carbohydrate chemical make-up and conformation, as revealed by advanced molecular spectroscopy with uni- and multivariate chemometrics. The biological and nutritional significance of these changes will be evaluated in further studies. The current study, for the first time, provided molecular structural information on transgenic *alfalfa* plants and insight into the impact of transgenes on protein and carbohydrate properties and their molecular structure.

## Figures and Tables

**Table 1:** Effect of double gene and single gene transformation on protein-related molecular spectral characterization of *alfalfa.*

*Alfalfa* Population	Protein Amide Height			Protein Amide Area				Protein Fine Structure		
	(Baseline ~1710–1487)			(Baseline ~1710–1487)				(Baseline ~1710–1487)		
	Amide I Height (~1650 cm^−1^)	Amide II Height (~1550 cm^−1^)	Ratio of Amide I/II Heights	Amide I Area	Amide II Area	Amide I and II Total Area	Ratio of Amide I/II Area	α-Helix Height (~1650 cm^−1^)	β-Sheet Height (~1596 cm^−1^)	Ratio of α-Helix/β-Sheet Height
*C1*	0.029	0.019	1.536	1.598 ^a,b^	2.197 ^a,b^	3.795 ^a,b^	0.725 ^b,c^	0.027 ^a,b^	0.027	0.983 ^b^
*Lc1*	0.024	0.015	1.533	1.261 ^b^	1.729 ^b^	2.990 ^b^	0.729 ^b,c^	0.023 ^b^	0.02	1.109 ^b^
*Lc3*	0.028	0.018	1.519	1.518 ^a,b^	2.041 ^a,b^	3.558 ^a,b^	0.744 ^a,b,c^	0.025 ^a,b^	0.023	1.088 ^b^
*Lc1C1*	0.029	0.019	1.582	1.658 ^a^	2.160 ^a,b^	3.817 ^a,b^	0.768 ^a^	0.029 ^a,b^	0.041	1.048 ^b^
*Lc3C1*	0.027	0.016	1.657	1.452 ^a,b^	2.017 ^a,b^	3.469 ^a,b^	0.718 ^c,d^	0.025 ^a,b^	0.024	1.045 ^b^
NT	0.029	0.025	1.497	1.579 ^a,b^	2.279 ^a^	3.858 ^a^	0.694 ^d^	0.027 ^a,b^	0.027	1.012 ^b^
ACGL	0.030	0.019	1.572	1.589 ^a,b^	2.122 ^a,b^	3.711 ^a,b^	0.750 ^a,b^	0.029 ^a^	0.022	1.319 ^a^
SEM	0.0014	0.0032	0.0386	0.0821	0.1133	0.1944	0.0074	0.0014	0.006	0.0302
*p* value	0.08	0.38	0.07	0.04	0.04	0.049	<0.01	0.04	0.29	<0.01
Contrast *p* value										
Single vs. Double	0.23	0.99	0.01	0.21	0.35	0.29	0.15	0.09	0.11	0.64
NT vs. Trans	0.34	0.02	0.08	0.32	0.03	0.09	<0.01	0.36	0.93	0.16
NT vs. Single	0.20	0.03	0.44	0.18	0.02	0.05	<0.01	0.15	0.63	0.14
NT vs. Double	0.81	0.04	0.01	0.79	0.13	0.33	<0.01	0.96	0.38	0.31
*C1* vs. *Lc*	0.08	0.63	0.83	0.05	0.03	0.04	0.21	0.13	0.52	<0.01
ACGL vs. Single	0.04	0.66	0.35	0.18	0.32	0.25	0.06	0.01	0.86	<0.01
ACGL vs. Double	0.30	0.67	0.31	0.74	0.80	0.77	0.47	0.21	0.16	<0.01

^a–d^ Means with different letters within same row differ (*p* < 0.05); SEM: Standard error of means; ACGL = AC-Grazeland; *C1* = Single transgenic *C1*; *Lc1* = Single transgenic *Lc1*; *Lc1C1* = Double transgenic *Lc1* + *C1*; *Lc3* = Single transgenic *Lc3*; *Lc3C1* = Double transgenic *Lc3* + *C1*; NT = Non-transgenic parent plant.

**Table 2:** Effect of double gene and single gene transformation on carbohydrate-related molecular spectral characterization of *alfalfa*.

*Alfalfa* Population	Structural Carbohydrates (StCHO) Profile				Cellulosic Compound Profile		Total Carbohydrate (TCHO) Profile						
	Peak 1 Height (~1410)	Peak 2 Height (~1370)	Peak 3 Height (~1240)	Total Area	Height (~1240)	Area	Peak 1 Height (~1153)	Peak 2 Height (~1080)	Peak 3 Height (~1020)	Peak 1 Area	Peak 2 Area	Peak 3 Area	Total Area
	Baseline				Baseline		Baseline			Baselines			
	~1487–1190				~1290–1190		~1190–880			~1190–1128	~1128–1055	~1055–880	~1190–880
*C1*	0.014	0.012	0.010	2.532	0.007	0.322	0.016 ^a,b^	0.052	0.064 ^a,b^	0.705	2.962	4.759 ^a^	8.426
*Lc1*	0.013	0.011	0.010	2.318	0.008	0.363	0.015 ^a,b^	0.053	0.064 ^a,b,c^	0.695	3.120	4.629 ^a,b^	8.444
*Lc3*	0.014	0.012	0.010	2.539	0.007	0.359	0.016 ^a,b^	0.054	0.066 ^a,b^	0.746	3.192	4.727 ^a^	8.665
*Lc1C1*	0.015	0.012	0.010	2.527	0.008	0.351	0.016 ^a,b^	0.054	0.064 ^a,b^	0.720	3.296	4.508 ^a,b^	8.524
*Lc3C1*	0.013	0.011	0.010	2.343	0.007	0.335	0.014 ^b^	0.047	0.055 ^b,c^	0.654	2.809	4.152 ^a,b^	7.614
NT	0.014	0.014	0.011	2.713	0.008	0.351	0.017 ^a^	0.053	0.068 ^a^	0.750	3.070	4.972 ^a^	8.810
ACGL	0.012	0.011	0.010	2.233	0.008	0.375	0.015 ^a,b^	0.051	0.051 ^c^	0.690	3.288	3.736 ^b^	7.713
SEM	0.0007	0.0009	0.0005	0.122	0.0004	0.0185	0.0007	0.0024	0.0028	0.0319	0.1369	0.2153	0.3808
*p* value	0.07	0.09	0.49	0.07	0.61	0.48	0.04	0.37	<0.01	0.34	0.13	<0.01	0.16
Contrast *p* value													
Single vs. Double	0.60	0.85	0.68	0.81	0.94	0.78	0.16	0.32	0.08	0.34	0.76	0.07	0.22
NT vs. Trans	0.54	0.02	0.05	0.03	0.42	0.78	0.07	0.51	0.09	0.15	0.97	0.05	0.21
NT vs. Single	0.45	0.03	0.10	0.06	0.48	0.87	0.24	0.79	0.33	0.32	0.89	0.25	0.47
NT vs. Double	0.78	0.03	0.05	0.04	0.45	0.69	0.02	0.28	0.02	0.08	0.91	0.01	0.08
*C1* vs. *Lc*	0.26	0.41	0.74	0.51	0.15	0.10	0.66	0.55	0.83	0.71	0.27	0.77	0.79
ACGL vs. Single	0.04	0.24	0.81	0.11	0.37	0.21	0.18	0.42	<0.01	0.50	0.22	<0.01	0.08
ACGL vs. Double	0.02	0.32	0.93	0.17	0.35	0.15	0.83	0.99	0.01	0.94	0.16	0.02	0.44

^a–d^ Means with different letters within same row differ (*p* < 0.05); SEM: Standard error of means; ACGL = AC-Grazeland; *C1* = Single transgenic *C1*; *Lc1* = Single transgenic *Lc1*; *Lc1C1* = Double transgenic *Lc1* + *C1*; *Lc3* = Single transgenic *Lc3*; *Lc3C1* = Double transgenic *Lc3* + *C1*; NT = Non-transgenic parentplant.

## References

[1] Jonker A., Gruber M.Y., Mccaslin M., Wang Y., Coulman B., Mckinnon J.J., Christensen D.A., Yu P. (2010). Nutrient composition and degradation profiles of anthocyanidin-accumulating Lc-*alfalfa* populations. Can. J. Anim. Sci..

[2] Jonker A., Gruber M.Y., Wang Y., Coulman B., Azarfar A., McKinnon J.J., Christensen D.A., Yu P. (2011). Modeling degradation ratios and nutrient availability of anthocyanidin-accumulating Lc-*alfalfa* populations in dairy cows. J. Dairy Sci..

[3] Ray H., Yu M., Auser P., Blahut-Beatty L., McKersie B., Bowley S., Westcott N., Coulman B., Lloyd A., Gruber M.Y. (2003). Expression of anthocyanins and proanthocyanidins after transformation of *alfalfa* with maize *Lc*. Plant Physiol..

[4] Peel G.J., Pang Y., Modolo L.V., Dixon R.A. (2009). The LAP1 MYB transcription factor orchestrates anthocyanidin biosynthesis and glycosylation in Medicago. Plant J..

[5] Hancock K.R., Collette V., Fraser K., Greig M., Xue H., Richardson K., Jones C., Rasmussen S. (2012). Expression of the R2R3-MYB transcription factor TaMYB14 from *Trifolium arvense* activates proanthocyanidin biosynthesis in the legumes *Trifolium repens* and *Medicago sativa*. Plant Physiol..

[6] Doiron K., Yu P., McKinnon J.J., Christensen D.A. (2009). Heat-induced protein structure and subfractions in relation to protein degradation kinetics and intestinal availability in dairy cattle. J. Dairy Sci..

[7] Xin H., Zhang X., Yu P. (2013). Using synchrotron radiation-based infrared microspectroscopy to reveal microchemical structure characterization: Frost damaged wheat vs. normal wheat. Int. J. Mol. Sci..

[8] Yu P., Christensen C.R., Christensen D.A., McKinnon J.J. (2005). Ultrastructural-chemical make-up of yellow-seeded (*Brassica rapa*) and brown-seeded (*Brassica napus*) canola within cellular dimensions, explored with synchrotron reflection FTIR microspectroscopy. Can. J. Plant Sci..

[9] Yu P. (2010). Plant-based food and feed protein structure changes induced by gene-transformation, heating and bio-ethanol processing: A synchrotron-based molecular structure and nutrition research program. Food Mol. Nutr..

[10] Yu P., Nuez-Ortín W.G. (2010). Relationship of protein molecular structure to metabolisable proteins in different types of dried distillers grains with solubles: A novel approach. Br. J. Nutr..

[11] Jackson M., Mantsch H.H. (1995). The use and misuse of FTIR spectroscopy in the determination of protein structure. Crit. Rev. Biochem. Mol. Biol..

[12] Yu P., Jonker A., Gruber M. (2009). Molecular basis of protein structure in proanthocyanidin and anthocyanin-enhanced *Lc*-transgenic *alfalfa* in relation to nutritive value using synchrotron-radiation FTIR microspectroscopy: A novel approach. Spectrochim. Acta A Mol. Biomol. Spectrosc..

[13] Wetzel D.L., Eilert A.J., Pietrzak L.N., Miller S.S., Sweat J.A. (1998). Ultraspatially resolved synchrotron infrared microspectroscopyof plant tissue in situ. Cell. Mol. Biol..

[14] Yu P. (2004). Application of advanced synchrotron radiation-based Fourier transform infrared (SR-FTIR) microspectroscopy to animal nutrition and feed science: A novel approach. Br. J. Nutr..

[15] Yu P., Block H., Niu Z., Doiron K. (2007). Rapid characterization of molecular chemistry, nutrient make-up and microlocation of internal seed tissue. J. Synchrotron Radiat..

[16] Fick G.W., Mueller S.C. (1989). *Alfalfa*-quality, maturity and mean stage of development. Inf. Bull..

[17] Yari M., Valizadeh R., Naserian A.A., Jonker A., Yu P. (2013). Protein molecular structures in *alfalfa* hay cut at three stages of maturity and in the afternoon and morning and relationship with nutrient availability in ruminants. J. Sci. Food Agric..

[18] Yu P. (2012). Short communication: Relationship of carbohydrate molecular spectroscopic features to carbohydrate nutrient profiles in co-products from bioethanol production. J. Dairy Sci..

[19] Yu P. (2005). Applications of hierarchical cluster analysis (CLA) and principal component analysis (PCA) in feed structure and feed molecular chemistry research, using synchrotron-based fourier transform infrared (FTIR) microspectroscopy. J. Agric. Food Chem..

[20] Yu P. (2005). Protein secondary structures (α-helix and β-sheet) at a cellular level and protein fractions in relation to rumen degradation behaviours of protein: A new approach. Br. J. Nutr..

[21] Yu P. (2008). Synchrotron-based microspectroscopic analysis of molecular and biopolymer structures using multivariate techniques and advanced multi-components modeling. Can. J. Anal. Sci. Spectrosc..

[22] Saxton A.M. (1998). A macro for converting mean separation output to letter groupings in PROC MIXED. Proceedings of the 23rd SAS Users Group International.

